# Dietary Fiber Intake Is Related to Skeletal Muscle Mass, Body Fat Mass, and Muscle-to-Fat Ratio Among People With Type 2 Diabetes: A Cross-Sectional Study

**DOI:** 10.3389/fnut.2022.881877

**Published:** 2022-05-31

**Authors:** Fuyuko Takahashi, Yoshitaka Hashimoto, Ayumi Kaji, Ryosuke Sakai, Yuka Kawate, Takuro Okamura, Hiroshi Okada, Noriyuki Kitagawa, Naoko Nakanishi, Saori Majima, Takafumi Osaka, Takafumi Senmaru, Emi Ushigome, Mai Asano, Masahide Hamaguchi, Masahiro Yamazaki, Michiaki Fukui

**Affiliations:** ^1^Department of Endocrinology and Metabolism, Graduate School of Medical Science, Kyoto Prefectural University of Medicine, Kyoto, Japan; ^2^Department of Diabetology, Kameoka Municipal Hospital, Kameoka, Japan

**Keywords:** dietary fiber intake, skeletal muscle mass, body fat mass, type 2 diabetes, muscle to body fat ratio

## Abstract

**Objectives:**

To investigate the relationship between dietary fiber intake and skeletal muscle mass, body fat mass, and muscle-to-fat ratio (MFR) among men and women with type 2 diabetes (T2D).

**Methods:**

This cross-sectional study involved 260 men and 200 women with T2D. Percent skeletal muscle mass (%) or percent body fat mass (%) was calculated as (appendicular muscle mass [kg] or body fat mass [kg]/body weight [kg]) × 100. MFR was calculated as appendicular muscle mass divided by body fat mass. Information about dietary fiber intake (g/day) was obtained from a brief-type self-administered diet history questionnaire.

**Results:**

Dietary fiber intake was correlated with percent body fat mass (*r* = –0.163, *p* = 0.021), percent skeletal muscle mass (*r* = 0.176, *p* = 0.013), and MFR (*r* = 0.157, *p* = 0.026) in women. However, dietary fiber intake was not correlated with percent body fat mass (*r* = –0.100, *p* = 0.108), percent skeletal muscle mass (*r* = 0.055, *p* = 0.376), and MFR (*r* = 0.065, *p* = 0.295) in men. After adjusting for covariates, dietary fiber intake was correlated with percent body fat mass (β = 0.229, *p* = 0.009), percent skeletal muscle mass (β = 0.364, *p* < 0.001), and MFR (β = 0.245, *p* = 0.006) in women. Further, dietary fiber intake was related to percent skeletal muscle mass (β = 0.221, *p* = 0.008) and tended to be correlated with percent body fat mass (β = 0.148, *p* = 0.071) in men.

**Conclusion:**

Dietary fiber intake was correlated with skeletal muscle mass, body fat mass, and MFR among women with T2D.

## Introduction

There are increasing the number of elderly people with type 2 diabetes (T2D) (1). Diabetes accelerates muscle catabolism *via* insulin resistance and attenuating insulin signaling (2), causing rapidly loss of muscle mass and strength (3). Furthermore, older people with T2D frequently have sarcopenia (4), which is defined as the loss of muscle mass, strength, and function related to aging (5). Muscles are the main organs responsible for glucose metabolism in the body (6). Therefore, it is a critical target for treatment of T2D. A tight relationship between muscle atrophy and high visceral fat exists (7, 8). Muscle atrophy is caused by proinflammatory cytokines such as tumor necrosis factor-α (TNF-α) and interleukin-6 (IL-6) secreted from fat cells enlarged by obesity (9). Further, fat accumulation increases the risk factor for cardiovascular diseases (10) and all-cause death (11). The muscle-to-fat ratio (MFR) is a marker of cardiometabolic conditions and chronic kidney disease among the elderly people (12, 13). MFR is a marker of sarcopenic obesity, which is defined as the coexistence of sarcopenia and obesity (14). Compared with sarcopenia alone, sarcopenic obesity is a greater risk of microvascular complications (15) and mortality (16, 17) among patients with T2D.

Dietary fiber intake can improve glycemic control and decrease hyperinsulinemia and plasma lipid concentrations in people with T2D (18). A high dietary fiber intake is reported to be associated with a low risk of all-cause death and cardiometabolic disease (19). This association seems to be mediated partly by the effect of dietary fiber on body mass (20). Dietary fiber delays the movement of food from the stomach to the intestines, therefore causing digestion and absorption of carbohydrates to be slowed and blood glucose levels to be raised slowly (21). Insulin is secreted when blood glucose levels rise, which functions to import glucose into cells; however, it also synthesizes and stores excess glucose as glycogen and triglycerides (22). Therefore, it is useful to slow the rise of postprandial blood glucose to prevent the fat accumulation. Previous studies reported that a high dietary fiber intake was related to a greater lean mass and a lower fat mass (23) and a decreased prevalence of sarcopenia (24). Moreover, a higher dietary fiber consumption was related to a lower visceral adiposity and multiple biomarker levels implicated in inflammation (25). However, the association between dietary fiber intake and body compositions, such as skeletal muscle mass, body fat mass, and MFR in people with T2D is unknown. Therefore, this cross-sectional study examined this relationship among men and women with T2D, individually.

## Materials and Methods

### Study Participants

The KAMOGAWA-DM cohort study, a prospective cohort study, has begun in 2014 and continues now (26). It enrolled outpatients in the Department of Endocrinology and Metabolism, Kyoto Prefectural University of Medicine Hospital (Kyoto, Japan), and the Department of Diabetology, Kameoka Municipal Hospital (Kameoka, Japan). Patients who completed the questionnaire from January 2015 to September 2021 were enrolled in the cross-sectional research. The exclusion criteria were follows: patients without T2D, those with incomplete answers to the questionnaire, and those without data about multifrequency impedance body composition analyzer findings. The present study was approved by the local research ethics committee (no. RBMR-E-466-6) and was performed in accordance with the Declaration of Helsinki. A written informed consent was gathered from all participants.

### Data Collection

Family history and duration of diabetes, the habit of smoking, and the habit of exercise were obtained using a standardized questionnaire. Participants were separated according to smoking status (smokers and non-smokers). Exercising regularly was determined as constantly playing some kind of sport > 1 times per week. Furthermore, venous blood samples were obtained from patients after an overnight fast, and the fasting plasma glucose, the hemoglobin A1c (HbA1c), creatinine (Cr), triglyceride, and high-density lipoprotein cholesterol levels were assessed. The estimated glomerular filtration rate (eGFR) was estimated from the Japanese Society of Nephrology equation, which is as follows: eGFR = 194 × Cr^–1^.^094^ × age^–0^.^287^ (mL/min/1.73 m^2^) (× 0.739, if woman) (27). Blood pressure was evaluated using the HEM-906 device (OMRON, Kyoto, Japan) in a quiet space after 5 min of rest. Data about the medications for diabetes, including sodium glucose cotransporter-2 (SGLT2) inhibitors, insulin, metformin, and antihypertensive drugs were gathered from the medical records. Hypertension was defined as usage of antihypertensive drugs and/or blood pressure of ≥ 140/90 mmHg.

### Definition of Percent Skeletal Muscle Mass, Percent Body Fat Mass, and Muscle-to-Fat Ratio

Body weight (kg), appendicular muscle mass (kg), and body fat mass (kg) were assessed *via* InBody 720, which is a multifrequency impedance body composition analyzer (InBody Japan, Tokyo, Japan) (28). Body mass index (BMI, kg/m^2^) was calculated by dividing body weight in kilogram (kg) by height in meters squared (m^2^). Ideal body weight (IBW) was regarded as height in meters squared multiplied by 22 (29). Percent body fat mass (%) was calculated as (body fat mass [kg]/body weight [kg]) × 100. Percent skeletal muscle mass was calculated as (appendicular muscle mass/body weight) × 100 (30). MFR was estimated as appendicular muscle mass divided by body fat mass (14).

### Data About Habitual Diet Intake

A brief-type self-administered diet history questionnaire (BDHQ) was administered to measure habitual food and nutrient intake during the preceding 1-month period (31). The validation of BDHQ has been reported previously (32, 33). The median (interquartile range) Pearson correlation coefficients between the dietary record and the BDHQ were 0.54 (0.45–0.61) in women and 0.56 (0.41–0.63) in men (32). Data about the intake of energy, fat, carbohydrate, protein, including animal and vegetable proteins, dietary fiber, including soluble and insoluble dietary fiber, and alcohol consumption as well as fiber were collected using the BDHQ. Energy intake (kcal/IBW/day), fat intake (g/IBW/day), carbohydrate intake (g/IBW/day), and protein intake (g/IBW/day), including animal protein intake (g/IBW/day) and vegetable protein intake (g/IBW/day) were estimated. The carbohydrate-to-fiber ratio was calculated as carbohydrate intake divided by fiber intake (34).

### Statistical Analyses

Data were presented as means [standard deviation (SD)] or frequencies of potential confounding variables. The characteristics of men and women differed; thus, we analyzed data according to sex.

A correlation analysis was performed using Pearson’s correlation coefficient. We analyzed the correlation between body composition and dietary fiber intake in younger (age < 60 years) and older adults (age ≥ 60 years), separately (35, 36). Moreover, we analyzed the correlation between body composition and soluble or insoluble dietary fiber intake, and the correlation between macronutrient intakes and body composition. Investigating the association of dietary fiber intake with percent skeletal muscle mass, percent body fat mass, and MFR, multiple regression analyses were conducted after adjusting for potential cofounders including age, duration of diabetes, energy intake, the habit of exercise, the habit of smoking, alcohol consumption, HbA1c and creatinine levels, treatment with metformin (37), insulin (38) and SGLT2 inhibitor (39). Statistical analyses were performed using JMP (version 13.2; SAS Institute Inc., Cary, NC, United States) and EZR (Saitama Medical Center, Jichi Medical University, Saitama, Japan) (40), a graphical user interface for R (The R Foundation for Statistical Computing, Vienna, Austria). *P*-values of < 0.05 were regarded statistically significant.

## Results

**FIGURE 1:**
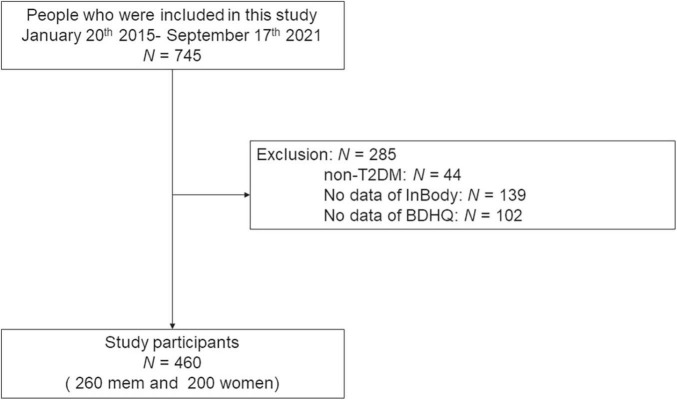
Study flow diagram for the registration of patients.

The current study included 745 people. Among them, 139 who did not carry out the bioelectrical impedance analysis test, 102 without the date of the BDHQ, and 44 without T2D were excluded. Therefore, 460 patients (260 men and 200 women) were finally involved in the present study (Figure 1).

Table 1 presents the clinical characteristics of the participants. The mean ages were 68.2 ± 10.6 years in men and 66.6 ± 10.8 years in women. The mean BMIs were 23.8 ± 3.7 kg/m^2^ in men and 25.2 ± 5.6 kg/m^2^ in women. The mean percent body fat mass and percent skeletal muscle mass were 25.2 ± 7.1 and 31.9 ± 4.4% in men and 34.8 ± 7.8 and 25.4 ± 3.9% in women, respectively. The mean MFRs were 1.5 ± 1.0 in men and 0.8 ± 0.3 in women.

**TABLE 1:** Clinical characteristics of study participants.

	Men *N* = 260	Women *N* = 200
Age (years)	68.2 (10.6)	66.6 (10.8)
Duration of diabetes (years)	15.5 (10.3)	14.3 (11.2)
Family history of diabetes (–/ +)	161/99	103/97
Height (cm)	167.0 (6.0)	153.1 (5.6)
Body weight (kg)	66.4 (11.2)	59.0 (12.9)
Body mass index (kg/m^2^)	23.8 (3.7)	25.2 (5.6)
Body fat mass (kg)	17.3 (7.3)	21.0 (8.7)
Percent body fat mass (%)	25.2 (7.1)	34.8 (7.8)
Appendicular muscle mass (kg)	20.9 (3.2)	14.7 (2.5)
Percent skeletal muscle mass (%)	31.9 (4.4)	25.4 (3.9)
Muscle-to-fat ratio	1.5 (1.0)	0.8 (0.3)
HbA1c (mmol/mol)	57.0 (13.0)	56.0 (12.8)
HbA1c (%)	7.4 (1.2)	7.3 (1.2)
Creatinine (umol/L)	85.6 (37.3)	62.5 (40.5)
eGFR (ml/min/1.73 m^2^)	67.5 (20.4)	71.2 (19.3)
Triglycerides (mmol/L)	1.5 (0.9)	1.4 (0.8)
HDL cholesterol (mmol/L)	1.5 (0.4)	1.6 (0.4)
Systolic blood pressure (mmHg)	133.1 (17.5)	134.1 (19.4)
Diastolic blood pressure (mmHg)	77.3 (12.0)	76.2 (12.5)
Antihypertensive drugs (–/ +)	111/149	98/102
Presence of hypertension (–/ +)	85/175	69/131
Insulin (–/ +)	196/64	150/50
Metformin (–/ +)	156/104	111/89
SGLT2 inhibitor (–/ +)	216/44	169/31
Habit of smoking (–/ +)	208/52	187/13
Habit of exercise (–/ +)	128/132	109/91

*Data were expressed as mean (standard deviation) or number. eGFR, estimated glomerular filtration rate; HDL, high-density lipoprotein.*

Table 2 presents the dietary intake of the participants. The mean dietary fiber intakes were 12.8 ± 5.8 g/day in men and 11.7 ± 4.8 g/day in women.

**TABLE 2:** Habitual diet intake of study participants.

	Men *N* = 260	Women *N* = 200
Total energy intake (kcal/day)	1917.4 (627.6)	1508.5 (543.7)
Energy intake (kcal/IBW/day)	31.3 (10.6)	29.3 (10.8)
Total protein intake (g/day)	78.2 (32.1)	67.9 (29.3)
Protein intake (g/IBW/day)	1.3 (0.6)	1.3 (0.6)
Protein intake per energy intake (%)	16.3 (3.4)	17.9 (3.6)
Animal protein intake (g/day)	47.7 (25.1)	42.6 (23.4)
Animal protein intake (g/IBW/day)	0.8 (0.4)	0.8 (0.5)
Vegetable protein intake (g/day)	30.5 (10.7)	25.2 (9.0)
Vegetable protein intake (g/IBW/day)	0.5 (0.2)	0.5 (0.2)
Total fat intake (g/day)	60.1 (22.8)	51.0 (22.6)
Fat intake (g/IBW/day)	1.0 (0.4)	1.0 (0.5)
Fat intake per energy intake (%)	28.4 (6.4)	30.2 (5.8)
Total carbohydrate intake (g/day)	237.6 (86.6)	189.4 (70.4)
Carbohydrate intake (g/IBW/day)	3.9 (1.5)	3.7 (1.4)
Carbohydrate intake per energy intake (%)	49.8 (9.2)	50.5 (8.1)
Dietary fiber intake (g/day)	12.8 (5.8)	11.7 (4.8)
Carbohydrate-to-fiber ratio	20.4 (7.7)	17.5 (5.8)
Alcohol consumption (g/day)	12.9 (23.4)	0.9 (4.0)

*Data was expressed as mean (standard deviation) or number. IBW, ideal body weight.*

**FIGURE 2:**
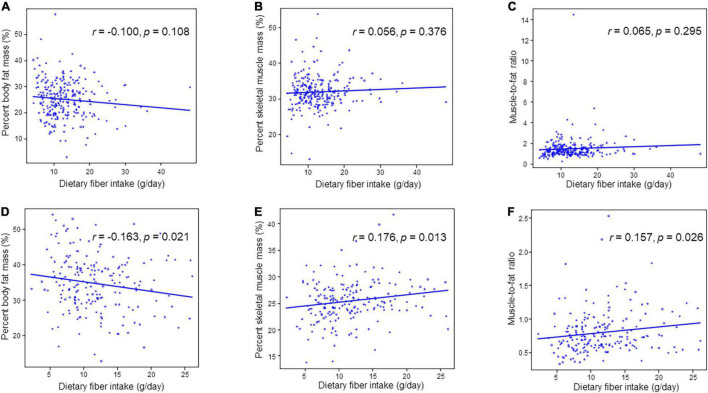
Correlation between dietary fiber intake and percent skeletal muscle mass, percent body fat mass or muscle-to-fat ratio. **(A)** Percent body fat mass in men. **(B)** Percent skeletal muscle mass in men. **(C)** Muscle-to-fat ratio in men. **(D)** Percent body fat mass in women. **(E)** Percent skeletal muscle mass in women. **(F)** Muscle-to-fat ratio in women.

Table 3 and Figure 2 depict the correlation between dietary fiber intake and clinical characteristics. Dietary fiber intake was correlated with percent body fat mass (*r* = –0.163, *p* = 0.021), percent skeletal muscle mass (*r* = 0.176, *p* = 0.013), and MFR (*r* = 0.157, *p* = 0.026) in women. However, dietary fiber intake was not related to percent body fat mass (*r* = –0.100, *p* = 0.108), percent skeletal muscle mass (*r* = 0.055, *p* = 0.376), and MFR (*r* = 0.065, *p* = 0.295) in men. HbA1c was not correlated with dietary fiber intake in men (*r* = 0.020, *p* = 0.755) and women (*r* = 0.023, *p* = 0.750). Dietary fiber intake was correlated with creatinine (*r* = –0.152, *p* = 0.032) and tended to be correlated with eGFR (*r* = 0.119, *p* = 0.092) in women. Dietary fiber intake was not associated with triglycerides (*r* = –0.089, *p* = 0.151 in men; *r* = 0.049, *p* = 0.496 in women) and HDL cholesterol (*r* = 0.060, *p* = 0.332 in men; *r* = 0.063, *p* = 0.381 in women). The correlation between dietary fiber intake and systolic blood pressure was significant in men (*r* = 0.127, *p* = 0.042).

**TABLE 3:** Correlation between dietary fiber intake and clinical characteristics.

	Men *N* = 260		Women *N* = 200	
	*r*	*p*	*r*	*p*
Age (years)	0.171	0.006	0.079	0.268
Duration of diabetes (years)	0.027	0.662	–0.048	0.500
Body weight (kg)	–0.020	0.755	–0.081	0.252
Body mass index (kg/m^2^)	–0.006	0.922	–0.126	0.076
Body fat mass (kg)	–0.086	0.166	–0.121	0.087
Percent body fat mass (%)	–0.100	0.108	–0.163	0.021
Appendicular muscle mass (kg)	0.052	0.409	0.057	0.425
Percent skeletal muscle mass (%)	0.055	0.376	0.176	0.013
Muscle-to-fat ratio	0.065	0.295	0.157	0.026

*Correlations were analyzed using the Pearson’s correlation coefficient.*

In younger men (*n* = 42), percent body fat mass (*r* = –0.343, *p* = 0.026) and MFR (*r* = 0.309, *p* = 0.047) were correlated with dietary fiber intake. Percent skeletal muscle mass tended to be correlated with dietary fiber intake in both younger (*n* = 40) and older women (*n* = 160), although these correlations was not statistically significant. Moreover, percent body fat mass (*r* = –0.154, *p* = 0.052) and MFR (*r* = 0.152, *p* = 0.056) in older women tended to be correlated with fiber intake (Supplementary Table 1).

In women, soluble dietary fiber intake was correlated with percent body fat mass (*r* = –0.159, *p* = 0.024), percent skeletal muscle mass (*r* = 0.186, *p* = 0.008), and MFR (*r* = 0.152, *p* = 0.032), and insoluble dietary fiber intake was correlated percent body fat mass (*r* = –0.160, *p* = 0.024), percent skeletal muscle mass (*r* = 0.175, *p* = 0.013), and MFR (*r* = 0.157, *p* = 0.027) (Supplementary Table 2).

Fat intake was not associated with percent body fat in both men (*r* = 0.003, *p* = 0.966) and women (*r* = –0.031, *p* = 0.663). Each macronutrient intake was not associated with MFR in both men and women (Supplementary Table 3).

To investigate the association between dietary fiber intake and percent body fat mass, percent skeletal muscle mass, and MFR, multiple regression analyses were performed (Table 4). In women, dietary fiber intake was correlated with percent body fat mass (β = 0.229, *p* = 0.009), percent skeletal muscle mass (β = 0.364, *p* < 0.001), and MFR (β = 0.245, *p* = 0.006) after adjusting for covariates. Dietary fiber intake was related to percent skeletal muscle mass (β = 0.221, *p* = 0.008) and tended to be correlated with percent body fat mass (β = 0.148, *p* = 0.071) in men.

**TABLE 4:** Multiple regression analysis on percent body fat mass, percent skeletal muscle mass, and muscle-to-fat ratio.

Men	Percent body fat mass (%)		Percent skeletal muscle mass (%)		Muscle-to-fat ratio	
	β	*p*	β	*P*	β	*P*
Dietary fiber intake (g/day)	0.148	0.071	0.221	0.008	0.073	0.381
Age (years)	–0.009	0.899	–0.220	0.003	–0.034	0.642
Duration of diabetes (years)	0.099	0.142	0.134	0.052	–0.012	0.863
Energy intake (kcal/IBW/day)	–0.084	0.321	–0.186	0.033	–0.018	0.832
Exercise*	0.038	0.524	–0.025	0.685	0.003	0.963
Smoking^†^	0.136	0.025	0.062	0.308	0.029	0.636
Alcohol consumption (g/day)	0.125	0.065	0.213	0.002	0.055	0.427
HbA1c (%)	–0.014	0.822	–0.057	0.387	–0.012	0.855
Creatinine (umol/L)	0.057	0.365	0.103	0.107	0.261	<0.001
Insulin treatment^‡^	0.123	0.058	0.041	0.532	0.116	0.079
SGLT2 inhibitor^§^	–0.227	<0.001	–0.110	0.083	–0.096	0.130
Metformin^| |^	–0.050	0.449	0.036	0.597	–0.037	0.585

**Women**	**Percent body fat mass (%)**		**Percent skeletal muscle mass (%)**		**Muscle-to-fat ratio**	
	**β**	***P***	**β**	***P***	**β**	***P***

Dietary fiber intake (g/day)	0.229	0.009	0.364	< 0.001	0.245	0.006
Age (years)	0.044	0.561	–0.147	0.056	–0.033	0.668
Duration of diabetes (years)	0.185	0.011	0.116	0.119	0.200	0.008
Energy intake (kcal/IBW/day)	–0.083	0.335	–0.235	0.008	–0.097	0.273
Exercise*	0.105	0.126	–0.042	0.545	0.082	0.242
Smoking^†^	0.053	0.450	0.096	0.185	0.051	0.482
Alcohol consumption (g/day)	0.014	0.842	0.008	0.907	0.064	0.374
HbA1c (%)	–0.174	0.013	–0.230	0.001	–0.144	0.044
Creatinine (umol/L)	–0.112	0.100	–0.029	0.677	–0.107	0.124
Insulin treatment^‡^	0.062	0.397	0.0003	0.997	0.034	0.655
SGLT2 inhibitor^§^	–0.148	0.034	0.021	0.767	–0.140	0.049
Metformin^| |^	–0.120	0.100	–0.150	0.044	–0.102	0.170

*IBW, ideal body weight.*

*Exercise status was defined as non-regular exerciser (= 0) or regular exerciser (= 1).

*^†^Smoking status was defined as non-smoker (= 0) or smoker (= 1).*

*^‡^Insulin treatment, insulin secretagogues, insulin sensitizers, and nutrient load reducers were defined as without (= 0) or with (= 1).*

*^§^ SGLT2 inhibitor was defined as not use of SGLT2 inhibitor (= 0) or use of SGLT2 inhibitor (= 1).*

*^| |^ Metformin was defined as not use of metformin (= 0) or use of metformin (= 1).*

## Discussion

To the best of our knowledge, the present study first investigated the association between dietary fiber intake and body composition, which included skeletal muscle mass, body fat mass, and MFR among men and women with T2D. Results suggested that dietary fiber intake was related to percent body fat mass, percent skeletal muscle mass, and MFR in women. However, the relationship between dietary fiber intake and body composition in men should be further investigated.

The correlation between dietary fiber intake and percent body fat mass, percent skeletal muscle mass, and MFR might be explained by the following:

High visceral fat and muscle atrophy are closely related (7, 8). Muscle atrophy is induced by inflammatory cytokines such as TNF-α and IL-6 secreted from fat cells enlarged by obesity (9). Protein kinase B signaling and nuclear factor κB *via* the secretion of transforming growth factor-β, IL-6 and TNF-α was associated with sarcopenia (41). On the other hand, dietary fiber delays the movement of food from the stomach to the intestines, therefore causing blood glucose levels to be raised slowly (21). Suppressing the rise in postprandial glycemia avoids excessive insulin secretion and prevents the fat accumulation (42). A high dietary fiber intake is associated with the reduction of insulin resistance and fat accumulation (23, 25). A high dietary fiber intake and low inflammatory marker levels were found to be associated with visceral adiposity, which might be a consequence of dietary fiber-induced improvement in insulin sensitivity (25). Insulin resistance causes muscle atrophy (3). In addition, dietary fiber is associated with low IL-6 and TNF-α levels (43). In this study, dietary fiber intake might have suppressed fat accumulation and inflammation; therefore, it was positively correlated with skeletal muscle mass.

Dietary fiber intake greatly influences the metabolic activity and composition of gut microbiome (23). Gut microbiota affects the progression of obesity in the host. Moreover, gut microbiota in obese mice differed from that in normal mice (44, 45), and this relationship was found in the general population (46). Some gut bacteria grow using dietary fiber and make short-chain fatty acids (SCFAs) (47). In an animal study, SCFAs (particularly butyrate) prevented the translocation of lipopolysaccharide, a potent inflammatory molecule made in the cell membrane of gram-negative bacteria (48). Therefore, muscle atrophy might also be suppressed because dietary fiber intake inhibited inflammation based on body fat mass. Moreover, butyrate, one of SCFAs, promotes inducing regulatory T cells, which have a central role in the suppression of inflammatory (49). Regulatory T cells are known to have a protective effect on muscle atrophy (50). Therefore, the anti-inflammatory effects of fiber prevent muscle atrophy through SCFAs. Therefore, dietary fiber intake prevents muscle atrophy. Moreover, energy metabolism and glucose homeostasis in the host are promoted by SCFAs made by these bacteria (51, 52). SCFAs inhibit fat accumulation in adipose tissues (53) and increase skeletal muscle mass (54). Dietary fiber preserves lean body mass and decreases adiposity by increasing the biosynthesis of mitochondria and the oxidation of fatty acids in skeletal muscle (55). SCFA receptors are present not only in adipose tissues but also in skeletal muscle. SCFAs, particularly acetic acid, are an activator of AMP-activated protein kinase. Further, they promote oxidation of mitochondrial lipids in skeletal muscle *via* AMP production, which would lead to prevent muscle atrophy (56, 57). This study found that dietary fiber intake was related to MFR in women. Moreover, it improved MFR because dietary fiber intake might have suppressed body fat mass and prevented muscle atrophy.

In this study, there was no sufficient relationship between dietary fiber and body composition in men. It is unclear why the associations between dietary fiber intake and body composition in men and women are different. The effect of dietary fiber is considered to be more significant in women than in men, this might be because women are more likely to accumulate body fat and to experience muscle atrophy than men (58). In fact, it has been reported that dietary fiber intake suppresses the body fat mass (59). However, this study showed that dietary fiber intake tended to be correlated with body composition in men, although it did not reach statistically significance. The multivariate analysis showed correlations between dietary fiber and body compositions in men. Thus, further studies must be conducted to validate this relationship.

The current study had some limitations. First, due to its cross-sectional nature, causal relationships could not be identified. In this study, fat intake did not associate with percent fat mass in both men and women, although fat intake had been reported to be associated with fat accumulation (60). People with high percent fat mass might have reduced fat intake. Second, only Japanese individuals were enrolled in the current study. Thus, the results might not be generalizable to individuals from other ethnic backgrounds. Third, the body composition of participants was assessed *via* multifrequency impedance body composition analysis, not dual-energy X-ray absorptiometry. However, multifrequency impedance body composition analyzer data has a strong correlation with dual-energy X-ray absorptiometry data (61). Fourth, the observed correlations were relatively weak, and thus, there is a possibility that these acceptances are limited. Moreover, the sample size of this study was relatively small; therefore, we need further research with more participants. The participants in this study were almost older patients (*n* = 218/260 in men, *n* = 160/200 in women); therefore, further research is needed, especially about younger patients. Fifth, dietary fiber intake of the excluded group is not known exactly; thus, it is possible that dietary fiber intake in exclusion group is lower than that in including group.

To conclude, the present study first showed that dietary fiber intake was related to skeletal muscle mass, body fat mass, and MFR in women with T2D. Dietary fiber intake is important for not only preventing fat accumulation but also maintaining skeletal muscle mass.

## Data Availability Statement

The raw data supporting the conclusions of this article will be made available by the authors, without undue reservation.

## Ethics Statement

The studies involving human participants were reviewed and approved by the Kyoto Prefectural University of Medicine. The patients/participants provided their written informed consent to participate in this study.

## Author Contributions

FT contributed to design of the work, analysis and interpretation of data, and written the manuscript. YH contributed to conception and design the work, acquisition, analysis and interpretation of data, and revising the manuscript. AK and RS contributed to conception, design the work, acquisition, curation data, and contributed discussion. YK, TOk, NK, TOs, NN, SM, TS, HO, EU, MY, and MA contributed to acquisition data and contributed discussion. MH contributed to design of the work, acquisition data and contributed discussion. MF contributed to conception and design the work, acquisition and interpretation of data, and revising the manuscript. All authors have read and agreed to the published version of the manuscript.

## Conflict of Interest

YH received personal fees from Novo Nordisk Pharma Ltd., Mitsubishi Tanabe Pharma Corp., Kowa Company Ltd., Sanofi K.K., Takeda Pharmaceutical Co., Ltd., Ono Pharmaceutical Co., Ltd., Daiichi Sankyo Co., Ltd., and Sumitomo Dainippon Pharma Co., Ltd., outside of the submitted work. HO received grant support from the Japan Society for the Promotion of Science, and personal fees from Daiichi Sankyo Co., Ltd., Takeda Pharmaceutical Co., Ltd., Sumitomo Dainippon Pharma Co., Ltd., Novo Nordisk Pharma Ltd., MSD K.K., Kyowa Hakko Kirin Company Ltd., Kowa Pharmaceutical Co., Ltd., Eli Lilly Japan K.K., Ono Pharmaceutical Co., Ltd., Kissei Pharmaceutical Co., Ltd., Sanofi K.K., and Mitsubishi Tanabe Pharma Corporation. NN received grant support from Japan Society for the Promotion of Science (JSPS KAKENHI grant numbers: 19K23999 and 20K16158) and the Japan Food Chemical Research Foundation, and personal fees from Novo Nordisk Pharma Ltd., and Kowa Pharmaceutical Co., Ltd. TOs received grants from Combi Corporation, and personal fees from Toa Eiyo Corp., Mitsubishi Tanabe Pharma Corp., Daiichi Sankyo Co., Ltd., Novo Nordisk Pharma Ltd., Nippon Boehringer Ingelheim Co., Ltd., Ono Pharmaceutical Co., Ltd., Kyowa Kirin Co., Ltd., Sumitomo Dainippon Pharma Co., Ltd., MSD K.K., Takeda Pharmaceutical Co., Ltd., Kowa Pharma Co., LTD., Eli Lilly Japan K.K., and AstraZeneca K.K., outside of the submitted work. TS received personal fees from Kyowa Hakko Kirin Co., Ltd., Astellas Pharma Inc., Mitsubishi Tanabe Pharma Co., Kowa Pharma Co., Ltd., Sanofi K.K., Taisho Toyama Pharma Co., Ltd., Kissei Pharma Co., Ltd., MSD K.K., Novo Nordisk Pharma Ltd., Ono Pharma Co., Ltd., Eli Lilly Japan K.K., and Takeda Pharma Co., Ltd., outside of the submitted work. EU received grant support from the Japanese Study Group for Physiology and Management of Blood Pressure, Astellas Foundation for Research on Metabolic Disorders (grant number: 4024), Japan Society for the Promotion of Science, Mishima Kaiun Memorial Foundation, and personal fees from Sumitomo Dainippon Pharma Co., Ltd., Mitsubishi Tanabe Pharma Corporation, Nippon Boehringer Ingelheim Co., Ltd., Sanofi K.K., Kowa Pharmaceutical Co., Ltd., Daiichi Sankyo Co., Ltd., Kyowa Hakko Kirin Co., Ltd., AstraZeneca K.K., Novo Nordisk Pharma Ltd., Ono Pharmaceutical Co., Ltd., Taisho Pharmaceutical Co., Ltd., Takeda Pharmaceutical Company Ltd., and MSD K.K., outside of the submitted work. Donated Fund Laboratory of Diabetes therapeutics is an endowment department, supported with an unrestricted grant from Taiyo Kagaku Co., Ltd., Taisho Pharmaceutical Co., Ltd., and Ono Pharmaceutical Co., Ltd. MH received grants from Yamada Bee Farm, Oishi Kenko Inc., Nippon Boehringer Ingelheim Co., Ltd., AstraZeneca K.K., and Ono Pharma Co., Ltd., and personal fees from Eli Lilly, Japan, Sanofi K.K., Sumitomo Dainippon Pharma Co., Ltd., Daiichi Sankyo Co., Ltd., Mitsubishi Tanabe Pharma Corp., AstraZeneca K.K., Ono Pharma Co., Ltd., and Kowa Pharma Co., Ltd., outside of the submitted work. MA received personal fees from Takeda Pharmaceutical Co., Ltd., Kowa Pharmaceutical Co., Ltd., AstraZeneca K.K., Ono Pharmaceutical Co., Ltd., Abbott Japan Co., Ltd., Novo Nordisk Pharma Ltd., Chugai Pharmaceutical Co., Ltd., and Sumitomo Dainippon Pharma Co., Ltd., outside of the submitted work. MY received personal fees from Sumitomo Dainippon Pharma Co., Ltd., Kowa Pharmaceutical Co., Ltd., Takeda Pharmaceutical Company Limited, Kyowa Hakko Kirin Co., Ltd., Kowa Company, Limited, Daiichi Sankyo Co., Ltd., Ono Pharmaceutical Co., Ltd., AstraZeneca PLC, and MSD K.K., outside of the submitted work. MF received grants from Eli Lilly, Japan, K.K., Nippon Boehringer Ingelheim Co., Ltd., Sanwa Kagagu Kenkyusho Co., Ltd., Oishi Kenko Inc., MSD K.K., Kowa Pharma Co., Ltd., Kissei Pharma Co., Ltd., Sumitomo Dainippon Pharma Co., Ltd., Ono Pharma Co. Ltd., Mitsubishi Tanabe Pharma Corp., Abbott Japan Co., Ltd., Daiichi Sankyo Co., Ltd., Johnson &amp; Johnson K.K. Medical Co., Astellas Pharma Inc., Kyowa Kirin Co., Ltd., Novo Nordisk Pharma Ltd., Yamada Bee Farm, Taisho Pharma Co., Ltd., Terumo Corp., Takeda Pharma Co., Ltd., Tejin Pharma Ltd., Sanofi K.K., Nippon Chemiphar Co., Ltd., and TERUMO CORPORATION, and personal fees from Astellas Pharma Inc., Nippon Boehringer Ingelheim Co., Ltd., Sanwa Kagaku Kenkyusho Co., Ltd., MSD K.K., Mochida Pharma Co., Ltd., Eli Lilly Japan K.K., Kissei Pharma Co., Ltd., AstraZeneca K.K., Mitsubishi Tanabe Pharma Corp., TERUMO CORPORATION, Daiichi Sankyo Co., Ltd., Bayer Yakuhin, Ltd., Takeda Pharma Co., Ltd., Teijin Pharma Ltd., Ono Pharma Co., Ltd., Taisho Pharma Co., Ltd., Kyowa Kirin Co., Ltd., Abbott Japan Co., Ltd., Sumitomo Dainippon Pharma Co., Ltd., Arkray Inc., Medtronic Japan Co., Ltd., Novo Nordisk Pharma Ltd., Kowa Pharma Co., Ltd., Nipro Corp., and Sanofi K.K., outside of the submitted work. The remaining authors declare that the research was conducted in the absence of any commercial or financial relationships that could be construed as a potential conflict of interest.

## Publisher’s Note

All claims expressed in this article are solely those of the authors and do not necessarily represent those of their affiliated organizations, or those of the publisher, the editors and the reviewers. Any product that may be evaluated in this article, or claim that may be made by its manufacturer, is not guaranteed or endorsed by the publisher.
