# Supplementary material for: Dietary Fiber Intake Is Related to Skeletal Muscle Mass, Body Fat Mass, and Muscle-to-Fat Ratio Among People With Type 2 Diabetes: A Cross-Sectional Study
Source: Front Nutr. 2022 May 31;9:881877. doi: 10.3389/fnut.2022.881877 (PMC9194895; doi:10.3389/fnut.2022.881877)
Supplement: Supplementary file 1 [file Data_Sheet_1.pdf]

Supplemental table 1. Correlation between dietary fiber intake and body composition according age

Men	Age <60<br>N = 42		Age ≥60<br>N = 218	
	r	*p*	*r*	*p*
Body weight (kg)	-0.127	0.424	0.029	0.670
Body mass index (kg/m^2)	-0.140	0.378	0.030	0.663
Body fat mass (kg)	-0.277	0.076	-0.029	0.667
Percent body fat mass (%)	-0.343	0.026	-0.061	0.372
Appendicular muscle mass (kg)	0.075	0.638	0.100	0.142
Percent skeletal muscle mass (%)	0.201	0.201	0.045	0.509
Muscle-to-fat ratio	0.309	0.047	0.041	0.547
**Women**	**Age <60<br>N = 40**		**Age ≥60<br>N = 160**	
	r	*p*	*r*	*p*
Body weight (kg)	-0.172	0.288	-0.206	0.796
Body mass index (kg/m^2)	-0.214	0.185	-0.076	0.340
Body fat mass (kg)	-0.071	0.665	-0.111	0.161
Percent body fat mass (%)	-0.134	0.411	-0.154	0.052
Appendicular muscle mass (kg)	0.199	0.217	0.071	0.370
Percent skeletal muscle mass (%)	0.301	0.059	0.151	0.057
Muscle-to-fat ratio	0.123	0.450	0.152	0.056

Correlations were analyzed using the Pearson’s correlation coefficient.

Supplemental table 2. Correlation between soluble or insoluble dietary fiber intake and body composition

Men<br>N = 260	Soluble dietary fiber intake (g/day)		Insoluble dietary fiber intake (g/day)	
	r	*p*	*r*	*p*
Body weight (kg)	-0.015	0.805	-0.029	0.646
Body mass index (kg/m^2)	-0.014	0.829	-0.121	0.847
Body fat mass (kg)	-0.091	0.145	-0.095	0.128
Percent body fat mass (%)	-0.103	0.097	-0.109	0.078
Appendicular muscle mass (kg)	0.051	0.416	0.051	0.415
Percent skeletal muscle mass (%)	0.048	0.442	0.067	0.285
Muscle-to-fat ratio	0.076	0.224	0.070	0.261
**Women**<br>N = 200	Soluble dietary fiber intake (g/day)		Insoluble dietary fiber intake (g/day)	
	r	*p*	*r*	*p*
Body weight (kg)	-0.078	0.271	-0.076	0.288
Body mass index (kg/m^2)	-0.127	0.074	-0.119	0.093
Body fat mass (kg)	-0.114	0.107	-0.116	0.103
Percent body fat mass (%)	-0.159	0.024	-0.160	0.024
Appendicular muscle mass (kg)	0.069	0.329	0.062	0.381
Percent skeletal muscle mass (%)	0.186	0.008	0.175	0.013
Muscle-to-fat ratio	0.152	0.032	0.157	0.027

Correlations were analyzed using the Pearson’s correlation coefficient.

Supplemental table 3. Correlation between macronutrient intakes and body composition

Men<br>N = 260	Percent body fat mass (%)		Percent skeletal muscle mass (%)		Muscle-to-fat ratio	
	r	*p*	*r*	*p*	*r*	*p*
Total energy intake (kcal/day)	-0.050	0.426	0.054	0.388	0.047	0.448
Energy intake (kcal/IBW/day)	-0.042	0.501	-0.013	0.838	0.038	0.541
Total protein intake (g/day)	-0.040	0.524	0.020	0.749	0.008	0.895
Protein intake (g/IBW/day)	-0.034	0.580	-0.029	0.642	0.002	0.979
Protein intake per energy intake (%)	-0.001	0.988	-0.035	0.575	-0.050	0.422
Animal protein intake (g/day)	-0.002	0.972	-0.009	0.891	-0.031	0.621
Animal protein intake (g/IBW/day)	0.002	0.978	-0.046	0.458	-0.035	0.573
Vegetable protein intake (g/day)	-0.114	0.066	0.080	0.198	0.097	0.117
Vegetable protein intake (g/IBW/day)	-0.109	0.080	0.019	0.761	0.089	0.154
Total fat intake (g/day)	-0.001	0.981	-0.006	0.918	0.011	0.856
Fat intake (g/IBW/day)	0.003	0.966	-0.059	0.345	0.004	0.945
Fat intake per energy intake (%)	0.096	0.124	-0.108	0.082	-0.066	0.292
Total carbohydrate intake (g/day)	-0.039	0.529	0.032	0.610	0.071	0.254
Carbohydrate intake (g/IBW/day)	-0.035	0.578	-0.026	0.679	0.064	0.305
Carbohydrate intake per energy intake (%)	-0.026	0.677	-0.007	0.906	0.068	0.276

Women<br>N = 200	Percent body fat mass (%)		Percent skeletal muscle mass (%)		Muscle-to-fat ratio	
	r	*p*	*r*	*p*	*r*	*p*
Total energy intake (kcal/day)	-0.037	0.604	0.028	0.699	0.042	0.557
Energy intake (kcal/IBW/day)	-0.015	0.829	-0.043	0.545	0.013	0.855
Total protein intake (g/day)	-0.091	0.199	0.010	0.887	0.096	0.177
Protein intake (g/IBW/day)	-0.074	0.298	-0.046	0.514	0.072	0.312

Protein intake per energy intake (%)	-0.144	0.042	-0.031	0.666	0.130	0.067
Animal protein intake (g/day)	-0.072	0.311	-0.048	0.502	0.078	0.270
Animal protein intake (g/IBW/day)	-0.059	0.403	-0.086	0.224	0.061	0.394
Vegetable protein intake (g/day)	-0.110	0.112	0.157	0.026	0.109	0.126
Vegetable protein intake (g/IBW/day)	-0.094	0.187	0.078	0.272	0.082	0.247
Total fat intake (g/day)	-0.031	0.663	-0.040	0.577	0.024	0.732
Fat intake (g/IBW/day)	-0.016	0.826	-0.093	0.189	0.002	0.973
Fat intake per energy intake (%)	-0.021	0.773	-0.128	0.071	-0.023	0.748
Total carbohydrate intake (g/day)	-0.015	0.831	0.082	0.248	0.020	0.776
Carbohydrate intake (g/IBW/day)	0.008	0.912	0.010	0.888	-0.009	0.899
Carbohydrate intake per energy intake (%)	0.070	0.326	0.110	0.121	-0.054	0.445

Correlations were analyzed using the Pearson's correlation coefficient. IBW, ideal body weight.
